# Acknowledgment of Invited Editors

**DOI:** 10.1128/mBio.00455-17

**Published:** 2017-04-04

**Authors:** Arturo Casadevall

## EDITORIAL

On behalf of the editors of *mBio*, I gratefully acknowledge the following individuals, who served as invited editors for the journal in 2016. While not members of the Board of Editors, invited editors serve an important role in the review process. Invited editors are experts in their fields of research who add an additional level of quality to the review process. An editor may assign a paper to an invited editor when he/she would like to have an additional expert opinion of the reviews or when the subject area falls outside the editor’s primary area of expertise.

The time and effort of the following experts in handling articles have been essential to ensuring the high quality of our publications, and their help is greatly appreciated.

Rama Rao Amara
Daniel Barber
Stephen J. Barenkamp
Ralph S. Baric
Michael P. Barrett
Dennis A. Bazylinski
Marcel A. Behr
Stephen M. Beverley
David C. Bloom
Elhanan Borenstein
Romain Briandet
Roland Brosch
C. Titus Brown
Dennis R. Burton
Mark J. Buttner
Ambrose Cheung
Eric A. Cohen
Alan Collmer
Laurie E. Comstock
Carolyn B. Coyne
Blossom Damania
Robert Daum
Edward F. DeLong
Sarah E. F. D’Orazio
Pieter C. Dorrestein
Adam Driks
Luis Enjuanes
Paul D. Fey
Kevin Foster
Eric O. Freed
Clay Fuqua
Lee Gehrke
Michael Gillings
Gustavo H. Goldman
Andrew Goodman
Ann Griffen
Christoph Grundner
David L. Gutnick
Anders P. Hakansson
J. Marie Hardwick
Ronald N. Harty
David E. Heinrichs
Jeffrey P. Henderson
Alexander R. Horswill
Kelly T. Hughes
Gregory B. Hurst
Joseph M. Hyser
Akiko Iwasaki
Babak Javid
Jorgen Johansson
Paul D. R. Johnson
Yoshihiro Kawaoka
Dan Knights
David M. Knipe
Arash Komeili
Anna Konovalova
Natacha Kremer
Nathaniel Roy Landau
Jean-Paul Latge
Ben-hur Lee
Jean Claire Lee
Stuart M. Levitz
Jaisri R. Lingappa
Peter N. Lipke
George Liu
Haoping Liu
Melissa Bruckner Lodoen
Jeremy Luban
Rebecca Marie Lynch
Ryszard Maleszka
Anthony W. Maresso
Jose L. Martinez
Denise Monack
Cesare Montecucco
David C. Montefiori
Lynda A. Morrison
James M. Musser
David D. Myrold
Michael Noel Neely
Stuart J. Neil
Hiroshi Nikaido
Victor Nizet
Himadri B. Pakrasi
Kelli L. Palmer
Pyong Park
John Parkinson
Everett Pesci
David A. Rasko
Jyothi Rengarajan
Federico Rey
Anthony R. Richardson
D. Ashley Robinson
Forest Rohwer
Eugene Rosenberg
Ilan Rosenshine
Philip C. Rosenstiel
Craig R. Roy
Tracy Ruckwardt
Natividad Ruiz
Peter Sarnow
Herbert P. Schweizer
Ganes C. Sen
Joshua Shaevitz
M. Sloan Siegrist
Thomas J. Silhavy
David Skurnik
Justin Sonnenburg
Michael N. Starnbach
Frank Stewart
Gisela Storz
Paul D. Straight
Gurol Suel
Sue A. Tolin
Ana Traven
Susannah Green Tringe
Meera Unnikrishnan
Susana T. Valente
Jesus Valenzuela
Marjan W. van der Woude
Jorge Eugenio Vidal
Alan W. Walker
Mark J. Walker
Digby F. Warner
Scott C. Weaver
Malcolm Whiteway
Steven S. Witkin
Alan J. Wolfe

